# Understanding the spatial pattern and determinants of Airbnb revenue through a spatial regression approach: Perspective from Indonesian cities

**DOI:** 10.1371/journal.pone.0333738

**Published:** 2025-10-10

**Authors:** Adiwan Fahlan Aritenang, Zahratu Shabrina

**Affiliations:** 1 Urban and Regional Planning Program, School of Architecture, Planning and Policy Development (SAPPD), Institut Teknologi Bandung (ITB), Bandung, Indonesia; 2 Department of Geography, King’s College London, London, United Kingdom; University of Naples Federico II: Universita degli Studi di Napoli Federico II, ITALY

## Abstract

Airbnb adoption is growing in Indonesian cities, yet little is known about how its spatial dynamics intersect with urban features and tourism economies in cities of the Global South. This study presents a systematic spatial analysis of Airbnb performance in Indonesia, with a focus on Jakarta and Bandung. Using detailed performance data from AirDNA, we employ spatial autocorrelation and spatial regression models, specifically the Spatial Lag Model (SLM) and Spatial Error Model (SEM), to investigate the potential impact of urban amenities on Airbnb revenue. Our findings reveal distinct city-specific dynamics: in Bandung, Airbnb revenue is positively associated with the presence of restaurants and hotels but negatively correlated with concentrated commercial centres such as shopping malls, reflecting the city’s culinary-driven tourism economy. In contrast, in Jakarta, Airbnb revenue is strongly linked to shopping centres and restaurants, while hotels show no significant influence, suggesting Airbnb operates within differentiated market niches. These results underscore the critical role of local context and associated development policies in shaping platform economies, demonstrating that Airbnb’s performance cannot be generalised across cities, even within the same country. By highlighting the association between spatial factors and short-term rental markets in Indonesia, this paper contributes to the broader debate on sustainable tourism and platform urbanism in the Global South.

## 1. Introduction

Airbnb is a peer-to-peer (P2P) platform that enables individuals to list their properties as short-term holiday rentals. This mechanism is not entirely novel, as the platform connects those needing accommodation (guests) with those offering properties for rent (hosts), thereby facilitating transactions. This process often relies on a trust mechanism based on reviews [1,2]. Various studies have examined the relationship between Airbnb and other socioeconomic factors, particularly housing and gentrification in diverse global contexts [3–8], as well as its competition with traditional hotels [9,10]. Previous research has also highlighted the challenge of Airbnb-led touristification, which is associated with tourism gentrification, leading to pressures on local activities and the displacement of long-term residents [11,12].

There are limited studies on P2P accommodation platforms and their implications for urban development in Indonesia and the Global South in general, although interest is growing. The Global South highlights the distinct perception of Airbnb in local contexts. For instance, in South Africa, the attractiveness of Airbnb relies on its accessibility and convenience in relation to tourism products [13], while in Malaysia, there are substantial requirements for high-quality accommodation services, with cleanliness, hygiene, safety, and prices among the main determinants of preference [14]. Furthermore, Malaysia launched government support to promote Airbnb during Visit Malaysia Year 2014, encouraging tourists to book accommodations via Airbnb. A pilot project involving 130 homestays was launched in Melaka to list their private homes on Airbnb [15]. In a study based in Brazil, reviews on platforms such as Airbnb tend to be more positive due to personal interactions between hosts and guests [16]. Previous studies also suggest that an increasing number of Airbnb listings are located near tourist attractions [14,17,18].

Despite the emergence of P2P accommodation platforms, there is a lack of urban studies on how localities determine Airbnb’s performance and revenue as a metric for success. Currently, there is a lack of studies to understand the determinants of P2P accommodation revenue and its implications in the Global South. Most studies on P2P accommodation are related to tourism [17,19], marketing [20], consumer behaviour [21], and taxes [22], and predominantly focus on cities in the global North. While most Airbnb research has focused on Western cities, our study expands the geography of the debate by demonstrating how platform urbanism unfolds in Southeast Asia. Also, by combining spatial autocorrelation with spatial regression models (SLM and SEM), we capture not only localised drivers of Airbnb performance but also spatial spillover effects across neighbourhoods, an analytical perspective often overlooked in tourism and platform economy studies. Using these specific insights into Airbnb revenue, the paper aims to advance our knowledge of the determinants (spatial, property, and neighbourhood) of P2P accommodation revenue in the global South context.

This study draws on detailed Airbnb and urban data from Jakarta and Bandung, the second and third-largest Airbnb markets in Indonesia after Bali. As of April 2020, Bali hosted 39,977 active listings, while Jakarta had 5,617 and Bandung had 1,761. Other notable markets include Surabaya (995 listings), Yogyakarta (792), and Central Lombok (463) [23]. Jakarta and Bandung provide a particularly valuable comparison: they are only about 180 km apart, connected by extensive transport infrastructure, including the newly built high-speed rail [24,25] and toll roads [26,27], and share close ties in the tourism sector, particularly in peer-to-peer accommodation. Both cities also serve as the functional cores of their metropolitan regions: Jakarta’s metro area comprises 13 cities/regencies with a population of 28 million, while Bandung’s includes four cities/regencies with 9 million residents. Indonesia thus presents an interesting context for studying Airbnb, as it combines one of the world’s fastest-growing tourism sectors with rapidly expanding urban regions where platform-based accommodations intersect with diverse urban functions, strong intercity connectivity, and contrasting tourism economies.

The structure of this paper is as follows. Section 2 presents the theoretical framework underlying the research. Section 3 presents the research design, including data and methodology. In Section 4, we present the spatial analysis and regression models. Section 5 presents our conclusions and contributions.

## 2. Literature review

Airbnb has been disrupting the hotel industry by allowing guests to substitute for hotels, especially from small hotels [9,28]. Despite uncertainties regarding the extent of Airbnb’s competition with hotels, recent studies have highlighted that Airbnb demand is high [29,30]. Airbnb is arguably more dynamic than hotels, one reason being the rigidity of hotel development permits, which restrict hotels to specific areas. This significantly influences hotels’ investment decision-making and determines the services offered in the hotel industry [31]. Xie and Kwok [30] found that Airbnb’s presence in the same neighbourhood as hotels negatively impacts hotel performance. Blal et al. [28] studied the longitudinal impact of Airbnb on hotel performance and found that Airbnb shows both supplementary and substitution effects. These findings suggest that Airbnb listings have a different impact on hotel performance. Others have argued that P2P accommodation would continue to curb hotel revenue growth but may reduce new demand for hotel construction, thereby reducing hotel overbuilding [32].

Airbnb also impacts the surrounding neighbourhoods where the listings are located. Intrinsically, property listing characteristics affect occupancy rates [6,33,34]. Popular Airbnb listings are often located in residential areas, especially those with various amenities such as restaurants and other food and beverage establishments [35]. In the Global North, there is also an indication that Airbnb can be associated with the increase in rent prices that may lead to gentrification, as found in the tourist city of Palma Old Quarter in Spain and the competition between short-term and long-term rentals in London [5,36].

Currently, there is a lack of studies on P2P platforms in the Global South, particularly in Indonesia. The online review by the World Economic Forum remains the only authoritative analysis on the topic in Indonesia [37]. There is high competition among providers of digital platforms, despite barriers such as limited financial technology adoption among users. Other barriers include Indonesia’s limited access to smartphones (approximately 23%) and low credit card usage (at 5%), which is significantly lower compared to Cash (51%) and debit cards (29%), despite being the primary mode of financial transaction on digital platforms [38]. Various fintech payment apps such as Gopay, TCash, Ovo and others target less than 40 million mobile payment users in Indonesia [38].

There has been a demand for regulations to respond to traditional hotel concerns on accommodation rent competition by the Indonesian Employers Association (APINDO). They have filed an objection due to the presence of Airbnb, based on the fairness of business activity (Putera, 2017). Furthermore, the Indonesian Hotel and Restaurant Association (PHRI) demanded that the Minister of Communications and Information Technology (MCIT) block Airbnb applications as local hotels have been struggling to compete [39]. This local conflict, brought about by Airbnb, has also been observed elsewhere [6–9,33]. Mehmed [40] suggests that an innovative policy can be acquired through a partnership with grassroots initiatives, a comprehensive approach to individual needs, and incremental modifications to existing policies. A wide range of recent econometric studies has examined the determinants of Airbnb listing prices and revenues. The study by Wang and Nicolau [41] employed ordinary least squares and quantile regression models across 33 cities globally to identify price determinants, including host characteristics, property features, amenities, geographical location, and the number of guest reviews. The study found that professional hosts, flexible policies, and quality amenities significantly increase prices, especially for higher-end listings.

While revenue determinants, as identified by Gibbs et al. [42] and Xie and Kwok [30], who applied a hedonic pricing model to Airbnb listings in Canada and the USA, found that physical characteristics, location, particularly transit accessibility, and host traits all influenced price. Using spatial analysis, there is evidence that professionalisation was a significant factor, with a small number of commercial operators generating a disproportionate share of revenue and an increase in the number of guest reviews [43]. Furthermore, using a hedonic longitudinal mixed-model econometrics model, Blal et al. [28] found that Airbnb’s lower price and dispersed price range would reduce hotel revenue (Revenue Per Available Room/RevPAR). These finding highlights substitution effects where Airbnb becomes a direct competitor rather than just a supplemental option.

Taken together, these studies demonstrate that Airbnb performance is shaped by a combination of listing characteristics, host behaviour, market professionalisation, and competition with traditional hotels. However, much of the existing evidence is concentrated in Global North contexts, where tourism patterns, urban forms, and regulatory frameworks differ significantly from those in emerging economies. What remains underexplored is how Airbnb revenue is embedded within the spatial and urban fabric of Global South cities, where rapid urbanisation, diverse tourism drivers, and uneven regulatory environments create distinct dynamics.

## 3. Research design

### 3.1 Data and study area

This study uses a multidimensional dataset to analyse the pattern and determinants of Airbnb revenue in Bandung and Jakarta. The data is gathered from various sources, as can be seen in Table 1.

**Table 1:** Data Sources and Details.

*No*	*Data*	*Source*	*Description*
*1*	Airbnb Data 2016–2020	AirDNA	The data used in this study consists of three parts: (1) data regarding property information, (2) the monthly data on the property listings, and (3) the number of guest reviews data. Information was also provided regarding listing title, average daily rate (ADR), average annual revenue, number of bookings, response rate, super-host status and overall rating.
*2*	Point of Interests Data	Locations Extracted from Open Street Map (available under Open Database License)	Tags used to include tourism attractions (attraction, artwork, museum, viewpoint); accommodations (hotel, hostel, motel, campsite), shopping (malls and shopping centre), parks, and restaurants generated using overpass turbo engine (http://overpass-turbo.eu/).
*3*	Bandung Boundary Data	Boundary Data Extracted from Open Street Map (available under Open Database License)	Administrative boundary of Bandung in neighbourhood level *(kelurahan)* generated using the overpass turbo engine (http://overpass-turbo.eu/) using the tags *“admin_level=7”* in the query.
*4*	Jakarta Boundary Data	Boundary Data Extracted from Open Street Map (available under Open Database License)	Administrative boundary of Jakarta in neighbourhood level *(kelurahan)* generated using the overpass turbo engine (http://overpass-turbo.eu/) using tags *“admin_level=7”* in the query.

The Airbnb data was obtained from AirDNA, www.airdna.com, which provided the performance of Airbnb listings and the number of guest reviews for both Jakarta and Bandung cities between 2016 and 2020. The data were purchased from the Airdna platform. Due to funding availability, the authors limited the study to the period mentioned above, as Airbnb operated from 2016 to the onset of large-scale social restrictions (*Pembatasan Sosial Berskala Besar*/PSBB) implemented in April 2020. AirDNA has been used extensively by researchers and policymakers to analyse Airbnb activities globally for various purposes, including comparing STR performance, such as revenue and occupancy analysis, which is not available in other datasets [41–43,44–46].

The monthly data used in our study encompassed observations from January 2016 to April 2020 and included data on property type, Average Daily Rate (ADR), number of reservations, reservation days and geolocation information. For Jakarta, there were a total of 414,000 monthly observation data points, with 91,832 observations that had an occupancy rate, indicating that 22% of the listings were actively used. For Bandung, there were a total of 101,000 monthly observation data points, with 30,474 observations that included occupancy rates, indicating that 30% of the listings were actively used. The guest review data presents individual listings with the recorded number of guest reviews from the renters. All the data and analysis employed in this study comply with the terms and conditions of the data source.

The study area for this paper is two cities in Indonesia, Bandung and Jakarta. Bandung is the capital of West Java province and the third most populous city in Indonesia. It has the highest economic growth in the region due to the rapid advancements in its commercial, industrial, and educational facilities [47]. Bandung population has reached approximately 2.5 million [48]. Bandung is located 150 km from Jakarta, the most populous city in Indonesia, defined as a first-level autonomous region that is quite similar to a province that used to be known as the Capital City Special Region (*Daerah Khusus Ibukota –* DKI) [49]. However, Jakarta is now stripped of the status of a capital city following President Joko Widodo’s plan to move the capital city to Borneo. Jakarta’s population reached 10 million in 2020, and the city is one of the most populated global megacities with rapid economic growth [50].

**Fig 1:**
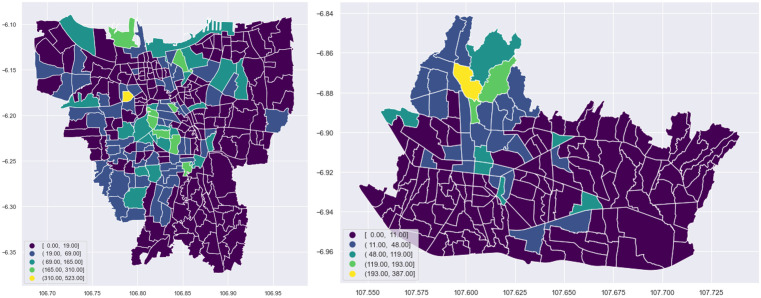
The Spatial Distribution of Airbnb Listings in Jakarta (Left) and Bandung (Right). [Contains information from OpenStreetMap and OpenStreetMap Foundation, which is made available under the Open Database License].

Fig 1 depicts the choropleth map of active Airbnb locations in Jakarta and Bandung based on the total Airbnb in 2020. We can see that Airbnb is unequally concentrated in certain areas, both in Bandung and Jakarta. Fig 1 shows a high number of Airbnb listings in Southwest and Central Jakarta, whilst the East and North parts of Jakarta represent the lowest number of Airbnb listings in the city. The north part of Bandung city is a cluster with a high number of Airbnb listings, whilst the south and east parts of the city are clusters of listings with a low number of Airbnb listings.

### 3.2 Methods and model specification

**Fig 2:**
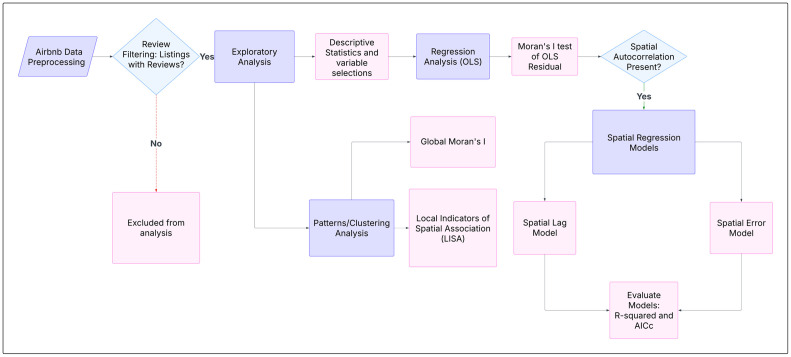
The Flowchart of Research Design.

In this paper, we conduct exploratory analysis and implement spatial regression models to examine patterns and determinants of Airbnb revenue in Bandung and Jakarta, Indonesia. The Airbnb data includes instant booking availability (to capture the flexibility of the host) and average daily rate (ADR) as a proxy for the price, capturing the revenue of Airbnb properties [9]. Fig 2 outlines the overall research design for analysing Airbnb data using various statistical and spatial modelling techniques. In the initial process, the data undergoes pre-processing followed by a data filtering step, as only Airbnb listings with at least one guest review will be included in the analysis, to determine active listings [4].

This study employs Spatial Lag Models (SLMs) and Spatial Error Models (SEMs) to account for spatial dependencies inherent in urban phenomena, such as Airbnb performance. Standard regression techniques assume that observations are independent, but in spatially structured urban environments, properties in proximity often influence each other’s outcomes. Using these complementary models allows us to isolate both direct and spillover effects, providing a more robust understanding of spatial patterns in Airbnb performance in Indonesia. The central hypothesis of this study is that Airbnb revenue is not uniformly distributed across the urban landscape but is instead strongly influenced by local urban amenities, tourism drivers, and spatial context.

#### 3.2.1 Spatial autocorrelation.

The Airbnb data are then explored using descriptive statistics, patterns, and clustering analysis for spatial autocorrelation, employing Moran’s I and Local Indicators of Spatial Autocorrelation (LISA) [51]. The Global and Local Moran’s I are methods for measuring spatial autocorrelation, aiming to provide insights into the nature of spatial dependence in spatial data. Spatial autocorrelation essentially shows correlation within variables across space, producing clustering of similar (positive spatial autocorrelation) or dissimilar (negative autocorrelation) values, typically displayed as clustering patterns on a geographical map [51]. The global Moran’s spatial autocorrelation measures the degree of spatial relationship or dependence in relation to *all* values in the dataset. In contrast, the local Moran’s spatial autocorrelation measures them individually in relation to their neighbourhood as established by the spatial weight matrix generally denoted as **W** [52]. The method allows us to examine if areas with high Airbnb revenue are surrounded by similar or dissimilar patterns.

#### 3.2.3 Linear Regression and Spatial Regression Analysis.

This study aims to examine the association between Airbnb revenue and other urban tourism contexts. The regression model uses the individual listing revenue as the independent variable in Indonesian Rupiah (IDR). The explanatory variables include points of interest such as shopping malls, tourist attractions, and restaurants. We also include control variables, such as parks, to capture the effect of proximity to open or public space on Airbnb accommodation revenue performance. Using Ordinary Least Squares (OLS) regression, we could estimate the parameters of a linear equation by minimising the sum of squared residuals. However, OLS assumes that observations are independent and normally distributed, an assumption that is often violated when dealing with spatial data due to the occurrence of spatial dependence or spatial autocorrelation, which can lead to biased model results [53,54]. One way to check the validity of the OLS result is to implement an analysis of the regression residuals. If spatial autocorrelation exists in the residuals, then we need to implement spatial regression models instead.

The model captures the effect of geographical proximity on Airbnb revenue by incorporating spatial dependencies into the model framework, with the spatial lag model (SLM) and the spatial error model (SEM) being the two primary approaches. SLM and SEM models are designed to account for the spatial dependencies that might occur when dealing with spatial datasets, where the neighbouring values influence variables in nearby locations. The spatial lag model incorporates spatial effects by including a spatially lagged dependent variable as an additional predictor. The following Equation (1) is the basic model of SLM:


y=ρWy+xβ+ε (1)

where *W*y is the spatially lagged dependent variable for the weights matrix W, *x* is a matrix of observations on the explanatory variables, ε is a vector of error and ρ is the spatial coefficient. Therefore, the SLM has the ability to capture interactions among neighbouring observations. Conversely, SEM corrects for spatial error dependence, ensuring that unobserved spatial effects do not lead to biases in the regression coefficients using the autoregressive error dependence as shown in Equation (2):


$$\begin{array}{c} y=x\beta +\varepsilon \\ \varepsilon =\rho W\varepsilon + \end{array}$$ (2)

Where Wε is the weight matrix, and is a vector of error terms. Using non-spatial regression and spatial regression, the study explores factors associated with Airbnb revenue in Indonesia to present a robust model to explain a geographically dependent phenomenon.

## 4. Results and discussion

### 4.1 Exploratory data analysis

To examine the Airbnb performance in the study area, we examine the Airbnb variables in Jakarta (split into West, Central, South, East and North Jakarta) and Bandung as can be seen in Table 2. On average, the average daily rate (ADR) per bedroom is found to be the highest in Central Jakarta at IDR 250,582 (USD 22.47) and the lowest in East Jakarta at IDR 200,641 (USD 16.92). South Jakarta has the highest number of active Airbnb listings, with 2824 listings, while East Jakarta has the lowest, with 530 listings. Bandung and Central Jakarta, with the highest ADR, also have the highest number of reviews. The response rates for all areas are similar, with approximately an 80% rate. The response rate refers to the percentage of any new inquiries and reservation requests that hosts have responded to within 24 hours in the past 30 days [55]. West Jakarta and Bandung have the highest percentages of superhosts, with 21.26% and 19.03%, respectively, referring to positive host performance, as indicated by receiving positive guest reviews, being responsive, and avoiding cancellations.

**Table 2:** Average Airbnb Metric Performance (2016 - 2020).

Areas	ADR* (IDR)	ADR (USD)	Response Rate	Number of Photos	Number of Reviews	Number of Superhost	Number of Listings	% of Superhost
West Jakarta	225202.5	19.31	85.3309	18.2722	11.7657	239	1124	21.26
Central Jakarta	250582.1	22.47	82.7853	17.594	14.343	260	1566	16.60
South Jakarta	243032.4	23.84	81.4502	16.1953	10.2443	483	2824	17.10
East Jakarta	200641.1	16.92	82.3109	15.3415	8.2572	95	530	17.92
North Jakarta	223362.9	18.48	81.6242	18.7516	10.2781	155	964	16.07
Bandung	211437.2	16.30	83.28335	15.478785	15.857836	395	2075	19.03

**Fig 3:**
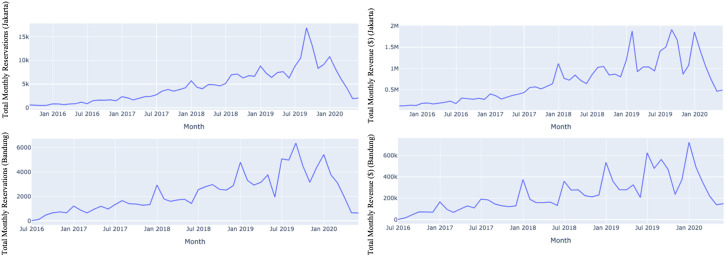
The Monthly Airbnb Reservations and Revenue in Jakarta (Top) and Bandung (Bottom).

Temporally, the overall monthly Airbnb reservations and revenues have been growing steadily, as shown in Fig 3. Despite the continuous increase, we can also observe some peaks and pits in the graphs that showcase the seasonality aspect of bookings. This aligns with other studies that have argued that the tourism and accommodation sector is highly seasonal [56].

This trend in Fig 3 shows a continuous increase except for 2020 when the COVID-19 pandemic hit globally. Lockdowns and restricted public gatherings were critical to avoid large-scale fatalities, contributing to restricted mobilities, hotel cancellations, and a loss in bookings, with a significant drop in domestic and foreign tourist arrivals in the tourism sector [56]. This might also affect Airbnb’s revenue in Indonesia; however, this is beyond the scope of our study.

### 4.2 Global and local spatial autocorrelation

Spatial autocorrelation could be defined as the absence of spatial randomness in that, for a given dataset, the *similarity in values* among observations relates to their *locational similarity*; hence relates to the target observation’s value with values in neighbouring locations for a specific variable. We detect the spatial pattern of Airbnb locations using Global Moran’s I statistic and Local Indicator for Spatial Autocorrelation (LISA). To calculate Moran’s I, we create the spatial weights matrix, *W*, using Queen’s method of contiguity weight. The result shows that the spatial distribution of high values in the dataset is more spatially clustered in Bandung than in Jakarta, as demonstrated by the Moran I p-value, 0.384 and 0.249, respectively.

**Fig 4:**
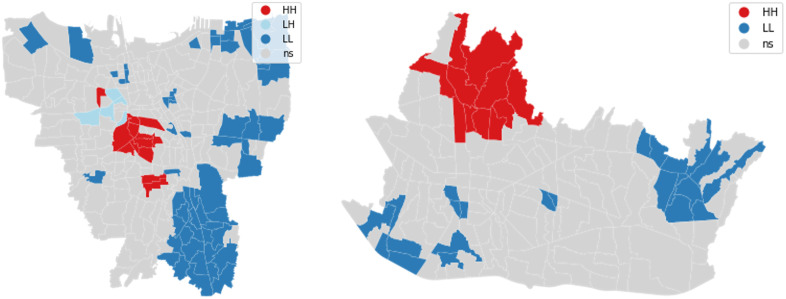
Local Spatial Cluster of Airbnb in Jakarta (Left) and Bandung (Right). [Contains information from OpenStreetMap and OpenStreetMap Foundation, which is made available under the Open Database License].

Using Airbnb data, this section maps the cluster/outlier type (COType) field that distinguishes between a statistically significant cluster of high values (HH), or so-called ‘hot spots’, representing areas where values at the site and surroundings are larger than average. On the other hand, clusters of low values (LL) are called ‘cold spots. The outliers, in which a high value is surrounded primarily by low values (HL), and those in which a low value is surrounded primarily by high values (LH), are labelled as non-significant (ns) at locations with non-significant p-values for the LISAs. From the analysis, we can see two types of clusters for Bandung and three types of clusters for Jakarta. The local spatial cluster of Airbnb revenue in Jakarta is concentrated on the city’s central, east, and north sides, as shown in Fig 4(left). For the hot spots (HH), there are ten urban villages where high-revenue Airbnb is surrounded by another high-revenue Airbnb, such as Duren Utara, Bendungan Hilir, Menteng, Setiabudi, Senayan, Semanggi, Kuningan, and Kalibata. These locations are part of/near the seven perceived hype neighbourhoods in Jakarta, as suggested by https://theculturetrip.com, a global culture website. Good accessibility to the tourism area or amenities could also be one of the reasons for this clustering, as the central location is also attributed to the ease of reaching these Airbnbs.

The local spatial cluster of Airbnb in Bandung dominates the city’s north, east and south sides as shown in Fig 4(right). North Bandung showcases clustering and hotspots for high-revenue Airbnb listings, dominated by hotels and restaurants in scenic view locations. North Bandung morphology is deeply rooted in the city’s historical development during the colonial era, with luxury housing owned by Dutch colonisers concentrated there [57]. This trend has continued in North Bandung, which is characterised by the presence of luxurious villas surrounded by various amenities and tourist attractions, whereas cold-spot areas are mostly industrial. On the other hand, land use in the hotspot areas is primarily commercial, with tourism and tourism amenities also present. The cold-spot areas are relatively far from tourist sites or amenities, such as restaurants and landmarks, and instead, most of the land in these areas is used for industry.

On the other hand, there is a different contour for the hot-spot and cold-spot areas; the altitude in the north is approximately 1050 meters above sea level (masl), while in the south, it is approximately 675 masl, which causes the weather in the north to be cooler than in the south.

### 4.3 Linear regression and spatial regression analysis

To examine the effect of spatial autocorrelation on Airbnb revenue distributions and investigate factors associated with Airbnb revenue, we apply both nonspatial and spatial regression models. We have included various possible determinants, including the density of restaurants, attractions, shopping malls, hotels, and parks. All the explanatory variables have been normalised by dividing the number of points of interest in a particular area by the total number of points in each city, thus showing proportion compared with the overall study area.

**Fig 5:**
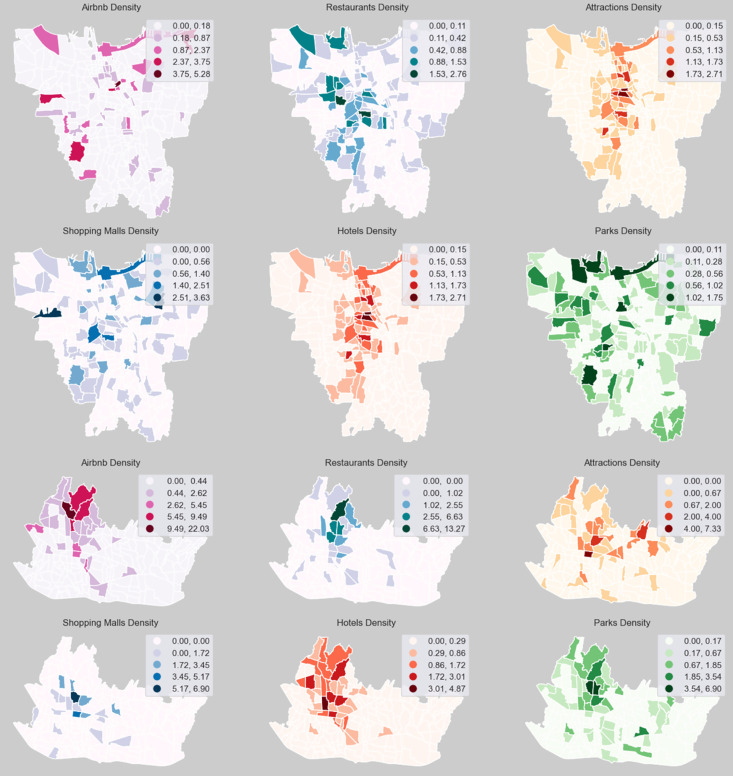
Spatial Distribution of Explanatory Variables in Jakarta (Top) and Bandung (Bottom). [Contains information from OpenStreetMap and OpenStreetMap Foundation, which is made available under the Open Database License].

Fig 5 shows the spatial distribution of each explanatory variable in Bandung and Jakarta. The figures confirm the previous section, which reveals hotspots of Airbnb listings in both cities. Jakarta exhibits a strong Northwest-Central-Southwest distribution for restaurants, a North-Central-South distribution for attractions and hotels, and a spatially distributed pattern of malls and parks. On the other hand, the variables in Bandung have a strong presence in the northern areas, which are well-known for having the most tourist destinations. It’s worth noting that Bandung tourism of social-cultural places, restaurants and hotels relies heavily on its natural beauty, such as mountain scenery, parks and diversity of food/culinary attractions. This finding contradicts findings in Chinese cities such as Shanghai, Beijing, and Hong Kong, where parks are the geographical factors for consumers to choose Airbnb, as argued by Wang and Wang [58].

**Fig 6:**
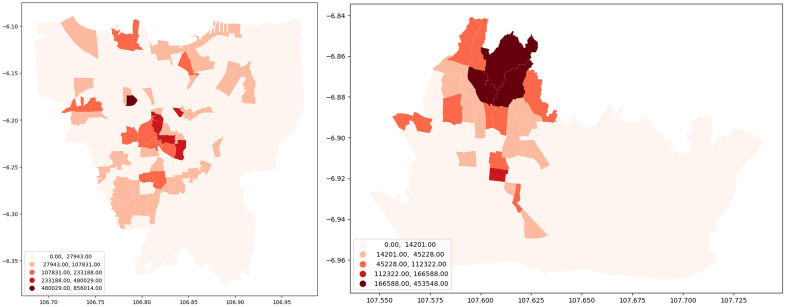
Airbnb Annual Revenue (USD) in Jakarta (Left) and Bandung (Right).

Table 3 presents the results from Ordinary Least Squares (OLS) and spatial regression models, specifically the Spatial Lag Model (autoregressive model) and the Spatial Error Model, for both Bandung and Jakarta. Initially, OLS was implemented to account for the association between Airbnb revenues in Jakarta and Bandung (Fig 6) and urban tourism components. Upon conducting Moran’s I test on the OLS residuals for both cities, the results show strong evidence of spatial autocorrelation (Moran’s I Bandung = 0.205; p-value <0.001 and Moran’s I Jakarta = 0.064; p-value <0.001). A statistically significant Moran’s I value of the OLS residuals indicates the presence of spatial autocorrelation in the residuals, meaning that the model could not fully account for spatial dependencies in the data, leading to biases in the model results [5]. This indicates that residuals are not randomly distributed and exhibit spatial patterns, which violates the assumption of random errors in the OLS model.

**Table 3:** OLS and Spatial Regression Model for Airbnb Revenue Determinants in Bandung and Jakarta.

	Ordinary Least Square (OLS)		Spatial Lag (SLM)		Spatial Error (SEM)	
	Bandung	Jakarta	Bandung	Jakarta	Bandung	Jakarta
**Hotels**	3,090*** (2,708)	3,401 (7,462)	9,169** (4,431)	2,679 (7,423)	7,721 (4,873)	4,356 [7,60]
**Shopping malls**	−5,689** (2813)	27,246*** (5,701)	−4,805 (2,687)	26,949*** (5,665)	−3,232 (2,771)	27,893*** (5,592)
**Tourist attractions**	3,903 (3,444)	−535 (3,367)	4,624 (3,290)	−539 (3,344)	5,720* (3,449)	−470 (3,288)
**Restaurants**	9847*** (2498)	34,976*** (7830)	9,003*** (2,387)	33,400*** (7,866)	8,115*** (2,405)	36,541*** (7,506)
**Parks**	−4,927 (3,973)	−14,635 (9,447)	−7,186 (3,827)	−14,499 (9,385)	−4,697 (4,53)	−15,306* (9,124)
**Constant**	3,090 (2,768)	3,675 (3,128)	1,592 (2,681)	3,147 (3,202)	4,687 (4,267)	3,096 (2,886)
**Spatial Lag**	–	–	0.408*** (0.093)	0.079 (0.083)	–	–
**Spatial Error (Lambda)**	–	–	–	–	0.415*** (0.099)	−0.106 (0.095)
**Numbers of Observations**	151	262	151	262	151	262
R-squared	0.149	0.113	–	–	–	–
Spatial Pseudo R-squared	–	–	0.190	0.118	0.142	0.113
AICc	7,285	12,901	7,270	12,902	7,275	12,900

Overall, the significance of the spatial error term (λ = 0.415***) further implies that unobserved spatial factors such as neighbourhood characteristics or local policies—also influence revenue, reinforcing the importance of spatial dependence in understanding Airbnb revenue.

### 4.4 Discussion

The OLS assumption of independently and identically distributed errors. In this case, spatial regression models, such as the Spatial Lag Model (SLM) or the Spatial Error Model (SEM), are more appropriate as they can explicitly account for spatial dependencies, thereby improving model accuracy and interpretation. The model results reveal notable differences in the association between tourism-related variables and Airbnb revenue across Bandung and Jakarta. Understanding the type of tourism associated with short-term rentals is crucial for developing effective policies, particularly in cities experiencing touristification, such as Bandung. Touristification is a term used to describe the *morphological transformation* of a community as a result of tourism-related activities, which could be induced by short-term rentals such as Airbnb [11,59].

In Bandung, a one-unit increase in hotel density is associated with an increase in Airbnb revenue locally and nearby areas due to spatial spillovers. For restaurants, the model shows a strong local effect and positive spillover. A unit increase in restaurant density is associated with ~9,003 increase of local revenue (annual USD), and an additional boost in neighbouring areas **—** this supports spatial clustering of food districts. In Jakarta, the number of restaurants exhibits a statistically significant positive effect, with a coefficient of 34,976, suggesting that restaurant density is associated with higher Airbnb revenues. This is present across all three models, including both non-spatial and spatial models. Shopping malls, on the other hand, display divergent effects: while they significantly contribute to the dependent variable in Jakarta (23,246), their impact in Bandung is negative (−5,689), indicating potential variations in consumer behaviour, urban tourism preferences, or spatial distribution of retail and leisure activities. The relatively low R-squared values (0.149 for Bandung and 0.113 for Jakarta) suggest that additional unobserved factors may influence the dependent variable, highlighting the limitations of OLS in capturing spatial dependencies within the dataset.

The tourist attractions variable is generally positive but has a statistically insignificant relationship with Airbnb annual revenue in Bandung across all models, with a weakly significant effect only in the Spatial Error Model (5,720*) for Bandung. This suggests that Airbnb listings located closer to tourist attractions in Bandung tend to generate higher revenues, likely due to the scenic view, cold weather and high accessibility to popular destinations that attract guests. In contrast, in Jakarta, the coefficient for tourist attractions is negative and consistently insignificant across all models, indicating that geographical proximity to tourist sites has no meaningful impact on Airbnb revenue in the city. These findings may reflect variations in how tourists engage with urban spaces in each city, as well as the density and distribution of attractions and listings.

A higher proportion of hotels and restaurants is positively associated with Airbnb revenue, whereas a higher proportion of shopping malls is negatively associated. In Bandung, both the spatial lag and error models indicate significant effects, suggesting that Airbnb listing locations are influenced by spatial dependence. The spatial lag regression reveals that hotels and restaurants continue to be significant positive factors for Airbnb revenue, with a notable feedback loop where neighbouring Airbnb revenues have a substantial impact on the dependent variable (p-value < 0.01). The spatial error model highlights that only the restaurant variable remains a significant positive predictor. The spatial error (lambda) also indicates an important relationship with the unexplained residual errors (p-value < 0.001). Spatial regression models provide a better explanation of the Airbnb revenue distribution in Bandung, as evidenced by higher R-squared values and lower AICc values. The AICc assesses the relative information loss due to overfitting and underfitting, commonly used for model selection in spatial models [60]. In Jakarta, shopping malls and restaurants consistently predict Airbnb revenue positively in spatial lag and error models. The spatial lag model is the better global model for Jakarta, with a higher R-squared, though AICc shows no significant improvement.

The findings align with a study by Pratminingsih et al. [61], which indicates that domestic tourists visit Bandung primarily to rest, relax, and engage in social interactions. Syarifuddin [62] highlights Bandung’s diverse attractions, including socio-cultural and natural environments. Tourism in Bandung encompasses a range of experiences, including culinary [63,64], shopping [65], environmental and educational [66,67], scientific [68], and other types of tourism, such as military caves and ghost tours [69]. The local tourism website lists neighbourhoods like Malabar (traditional food restaurants and cafes), Braga (a historical street with old buildings, shops, and cafes), North Bandung and Lembang (natural scenic areas with various activities), Cihampelas Street (Jeans Street), and Riau Street area (distros and restaurants). North Dago offers dining experiences with mountainous views.

In Jakarta, shopping malls and restaurants are significant positive predictors of Airbnb revenue in spatial lag and error models, indicating that retail centres should be considered tourism destinations. Large-scale mall development expanded significantly between 2010 and 2015 [70], shifting from purely shopping centres to mixed-use complexes with offices and apartments to attract a broader range of visitors. The early 2000s saw 41 predominantly regional shopping centres, while the early 2010s saw 63 new centres, 34.29% of which were specialised (trade centres), with convenience shopping centres emerging [71]. Shopping centres in peri-urban areas have evolved into large-scale, agglomerated hubs featuring food and entertainment centres that are frequented by customers. These centres enhance customer connectivity through accessible information like websites, audio-visual content, online applications, and self-service terminals [72].

These results align with studies by Fithriah, Susilowati, and Rizqihandari [73], which show that Jakarta’s tourists are mainly individual mass local tourists who plan their routes independently or with travel agents (48.98%). They often visit single attractions or prefer dispersed and adjacent sites. Nugraha et al. [74] found that 35% of tourists are interested in art and culture, natural scenery (35%), and study and research (15%). Susilowati [75] noted that Jakarta’s attractions are accessible by road and dispersed throughout the city, with 43.87% of visitors using private vehicles, as explorers visit multiple sites. In contrast, public transport is used by individual mass tourists, typically making single-point visits.

Overall, this study provides a spatially grounded analysis of Airbnb performance in Jakarta and Bandung, revealing how local urban and tourism contexts shape short-term rental revenues in distinct ways. By employing spatial econometric models (SLM and SEM) and focusing on key urban features such as restaurants, hotels, and shopping centres, the research demonstrates that Airbnb’s economic impact is highly place-specific: Bandung’s revenues are closely tied to culinary and hotel clusters, whereas Jakarta’s are linked to shopping centres and restaurants, diverging from traditional hotel markets. These findings underscore the significance of considering spatial dependencies and local context when evaluating platform-based tourism in the Global South, providing both theoretical insights into platform urbanism and practical guidance for sustainable urban and tourism planning in rapidly evolving cities.

## 4. Conclusion

Overall, this paper has examined the spatial and economic determinants of Airbnb revenue using Bandung and Jakarta as case studies. The findings demonstrate that Airbnb operates differently across the two cities, influenced by distinct tourism and economic drivers. Bandung’s Airbnb revenue is closely associated with cultural and culinary clusters, while in Jakarta it aligns with urban tourism and shopping centres, reflecting city-specific patterns of demand. These results underscore the importance of understanding local contexts when assessing platform-based accommodations and highlight how revenue can serve as a comprehensive metric for performance. More broadly, the study provides a nuanced perspective on how Airbnb interacts with other tourism elements, showing that tourists in Global South cities seek diverse, location-specific experiences.

The study has two key policy implications. First, regulations for short-term rentals (STRs) such as Airbnb should be tailored to local conditions rather than applying uniform policies nationwide. Our findings reveal that unobserved spatial factors, including neighbourhood characteristics and local development policies, are potential influential factors on revenue. Place-based policies, guided by differences in local endowments, institutions, and culture, as proposed by Barca et al. [76], can help reduce conflicts between businesses and urban developments. For example, high-revenue areas in Jakarta are tied to urban tourism, whereas in Bandung, revenue clusters around cultural and scenic zones in the northern part of the city, where hotels, art spaces, museums, and restaurants are located.

Second, sustainable tourism strategies must extend beyond popular destinations to include surrounding neighbourhoods. Policies for peer-to-peer accommodations should consider economic, social, and environmental dimensions to support local communities and preserve urban developments. In Bandung, certain tourism zones are under ecological pressure, highlighting the need for careful planning to balance tourism growth with environmental sustainability. Supporting such practices can both protect communities and enhance Airbnb revenue by maintaining attractive and resilient destinations. Overall, this study has answered a fundamental question that investigates the spatial and economic factors driving the performance of P2P accommodations.

Nonetheless, this study has limitations. The analysis uses pre-COVID-19 data, leaving the pandemic’s impact on Airbnb revenue unaccounted for. Future research could extend the temporal range to capture longer-term dynamics. Additionally, the study focuses on tourism-related and amenity variables aggregated at the neighbourhood level, without considering intrinsic listing characteristics such as host profiles, property features, or natural environmental factors (e.g., topography, scenic views, climate), which have been shown to influence accommodation performance [77,78]. Incorporating these factors in future work could provide a more detailed understanding of individual listing performance and offer deeper insights into the mechanisms driving Airbnb revenue.

In conclusion, this study contributes to global debates on platform urbanism and sustainable tourism by demonstrating how local context, spatial dependencies, and urban amenities jointly shape Airbnb performance in rapidly urbanising cities of the Global South. It highlights the importance of place-based policies and sustainable planning approaches, providing lessons that extend beyond Indonesia to other emerging tourism markets worldwide.
